# Does Training Background Influence Outcomes after Coronal Scalp Incision for Treating Craniomaxillofacial Injuries?: A German Pilot Study

**DOI:** 10.1007/s12663-023-01860-4

**Published:** 2023-02-02

**Authors:** Poramate Pitak-Arnnop, Keskanya Subbalekha, Chatpong Tangmanee, Nattapong Sirintawat, Jean-Paul Meningaud, Andreas Neff

**Affiliations:** 1 Department of Oral and Craniomaxillofacial Plastic Surgery, UKGM GmbH, University Hospital Marburg, Faculty of Medicine, Philipps-University of Marburg, Baldingerstr., 35043 Marburg, Germany; 2 Department of Oral and Maxillofacial Surgery, Faculty of Dentistry, Chulalongkorn University, 34 Henri Dunant Road, Bangkok, Pathum Wan 10330 Thailand; 3 Department of Statistics, Chulalongkorn University Business School, 254 Phyathai Road, Bangkok, Pathum Wan 10330 Thailand; 4 Department of Oral and Maxillofacial Surgery, Faculty of Dentistry, Mahidol University, 6 Yothi Road, Bangkok, Ratchathewi 10400 Thailand; 5 Department of Plastic, Reconstructive, Esthetic and Maxillofacial Surgery, Faculty of Medicine, Henri Mondor University Hospital, AP-HP, University Paris-Est Créteil Val de Marne (Paris XII), 1 rue Gustave Eiffel, 94000 Créteil, France

**Keywords:** Coronal incision, Complication, Craniomaxillofacial injury, Surgical education

## Abstract

**Objectives:**

To examine outcomes of the coronal scalp approach to craniomaxillofacial (CMF) fractures performed by oral-maxillofacial or craniofacial plastic surgery residents (OMFS/CFPS-Rs) *vs.* trauma surgery residents (TS-Rs), and to determine differences in treatment outcomes between both operator groups.

**Methods:**

This retrospective cohort study enrolled a sample of CMF fracture adult patients treated via the coronal approach in a German level one trauma center during a two-year interval. The predictor variable was training background (OMFS/CFPS-Rs *vs.* TS-Rs; each *n* = 5). All trainees must assist in ≥ two surgeries before self-performance. The main outcomes were length of hospital stay (LHS) and coronal flap-related complications (CFRCs). Appropriate statistics were computed at α = 95%.

**Results:**

Of the 97 patients identified during the study period; 71 of whom (19.7% females; mean age, 40.2 ± 15.2 years; 46.5% operated by TS-Rs; 38% combined upper and midfacial fractures) met the inclusion criteria. Operative time, LHS, CFRCs, readmission rates, and post-discharge emergency room visits were not significantly different between the trainee groups. 60% of CFRCs were visible/unfavorable or hypertrophic scar with/without alopecia. The number needed to treat of short LHS was 44 (95% confidence interval [CI], 3.9 to 4.8), the number needed to harm of CFRCs was 14 (95% CI, 3.6 to 7.4), i.e., the likelihood to be helped or harmed was 0.32.

**Conclusions:**

Coronal flap raising by OMFS/CFPS-Rs does not appear beneficial over that by TS-Rs in terms of LHS and CFRCs evaluated until postoperative month six. Trainees from any surgical specialties could gain partial independence from skilled surgeons in CMF trauma “sub-steps” and favorable clinical outcomes. Further studies in a larger sample cohort are required to confirm this pilot data.

## Introduction

The coronal approach to neurosurgical procedures was initially described by Hartley and Kenyon, and Babcock in early 1900. Its popularity among craniomaxillofacial (CMF; oral-maxillofacial [OMF]) and craniofacial plastic surgeons was championed by Paul Tessier, the father of modern craniofacial surgery. It has been considered as a standard approach to complex CMF deformities, craniotomy, harvesting of calvarial bone (e.g., for orbital repair) and temporal fascia, access to the mandibular condylar region, and forehead rejuvenation [1].

One mission of academic institutions is education of students and trainees who are also involved in patient care throughout hospitalization. While medical specialty education focuses on decision making, drug prescription, and counseling, surgical education additionally requires operative skill development. Recently, the treatment outcome has attracted much attention and been recognized as hospital’s and/or physician’s success which could affect reimbursement and reputation of both parties [2]. Hence, patient-orientated education should be monitored as, on one hand, a part of education, and, on the other hand, the quality of patient care.

Germany has encountered hidden dilemmas that only 8.3% of German hospitals own a CMF department that is directly responsible for CMF trauma care, and CMF education varies from one university to another [3, 4]. To promote holistic patient care, many hospitals hire a full- or part-time CMF surgeon in their trauma department for both CMF trauma education and patient care. Medical students and trainees in other specialties can, therefore, assist in CMF operations, or even perform a “sub-step” under close supervision. However, it remains unstudied whether this hospital’s arrangement model achieves good patient care.

The main purpose of this study was to examine treatment outcomes relative to the coronal scalp approach to CMF injuries, performed by oral-maxillofacial or craniofacial plastic surgery residents (OMFS/CFPS-Rs) *vs.* trauma surgery residents (TS-Rs). We also sought to determine whether treatment outcome differences existed between the operator groups. The null hypothesis was that there would be no difference in length of hospital stay (LHS) and coronal flap-related complications (CFRCs), when the flap was performed by OMFS/CFPS-Rs *vs.* TS-Rs. If the findings of this study favored the outcomes in the OMFS/CFPS-Rs group, the surgical “sub-step” education based on the above-mentioned integrative model of CMF surgeons in trauma units should be terminated, i.e., the coronal flap (and probably, any other “sub-steps”) ought to be performed by CMF surgeons or OMFS/CFPS-Rs only. We supposed that adequate basic knowledge, with exposure to a high volume of CMF trauma could help trainees perform their own sub-steps during the training period with favorable outcomes [5]. The specific aims were 1) to identify a sample of coronal flaps elevated by OMFS/CFPS-Rs and TS-Rs, 2) to document the type and frequency of CFRCs, and 3) to compare treatment outcomes between the trainee groups. We aimed to provide the 2011 Oxford Centre for Evidence-Based Medicine (OCEBM)’s level of evidence **“3”** and recommendation grade **“B”** at the study end.

## Materials and Methods

### Study Design and Sample Description

We designed and implemented a retrospective cohort study enrolling a sample of CMF fracture patients (aged ≥ 18 years) undergoing fracture repair via the coronal flap during a two-year interval. The study took place at a German level one trauma center with a main hospital and multiple neighboring hospitals within its networks. This hospital has faculty surgeons (including trauma surgeons, neurosurgeons, plastic surgeons, and a CMF surgeon), trauma surgery residents/fellows, and plastic and CMF surgery residents.

Trainees were included as study subjects if they 1) were third-to-fifth-year residents in CMF or craniofacial plastic or trauma surgery training (i.e., postgraduate year [PGY] three to five), or trauma surgery fellows (i.e., PGY six or more; i.e., board-certified trauma surgeons pursuing a subspecialty in trauma surgery), 2) assisted in ≥ two coronal flap raising, performed by the first author (P.P.), and 3) prepared the flap until the fractures could be visualized. The term “OMFS/CFPS-Rs” means 1) CMF residents, or 2) craniofacial plastic surgery residents whose operating catalog (logbook) consisted of ≥ 25% CMF procedures (i.e., pediatric/adult craniofacial surgery, head and neck reconstructive surgery, or reconstructive facial skeletal surgeries).

Exclusion criteria were (1) procedures that trainees cannot complete by themselves and needed help from the primary author (P.P.), (2) rotating plastic surgery residents from other training programs without CMF and/or had < 25% CMF procedures in their logbook, (3) procedures with higher operative risks, i.e., in patients with the American Society of Anesthesiologists (ASA) physical status level III to V, (4) procedures in patients with underlying diseases that may interfere with wound healing, e.g., diabetes mellitus, and (5) complex CMF trauma surgeries, for example, combined with neurosurgical interventions.

After obtaining the institutional review board approval, the Declaration of Helsinki, and the ICMJE and STROBE guidelines were followed throughout the study. Patients and trainees gave prospective consent for their anonymous data in future research. Some aspects of our results can be personally tracked and may influence on the trainee’s reputation. To conceal the identity and confidentiality of trainees participating in this study, we omit herein the exact time and place of the study.

### Surgical Technique and Postoperative Care

The coronal flap was raised in a standard fashion [1, 6–8]. After injection of a local anesthetic with vasoconstrictor and making an incision without the use of hemostatic clips (Raney clips), the flap was elevated atop the pericranium with finger or blunt periosteal elevators, or by back cutting with a scalpel. The plane of dissection along the lateral aspect of the skull was just superficial to the temporal muscle; hence, the facial nerve branches were included within the flap and would not be injured during the surgery [8].

**Fig. 1:**
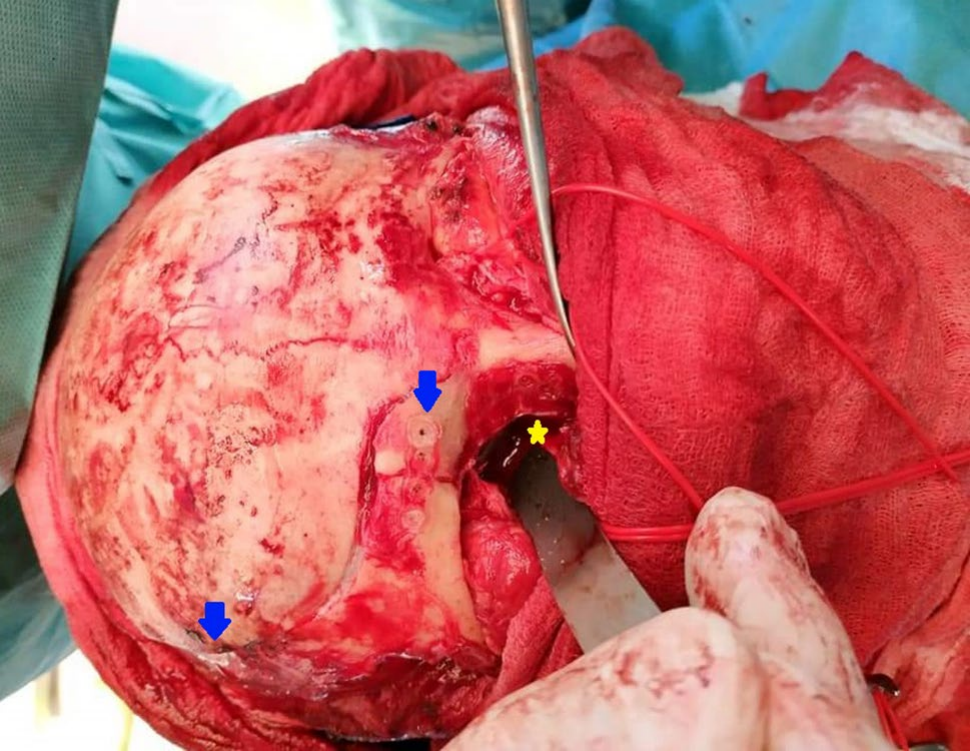
Intraoperative photograph showing frontotemporobasilar fractures repair via the coronal incision, including resorbable osteofixation (*SonicWeld* Rx®, KLS Martin, Tuttlingen, Germany; *blue arrows*) and orbital wall reconstruction using non-porous polydioxanone sheets (PDS® foil, Johnson&Johnson Medical GmBH, Ethicon, Norderstedt, Germany; *yellow asterix*)

To reduce risks of frontal sensory nerve injury, we pricked the areas supposed to be the location of frontal sensory nerves with needles, i.e., supratrochlear and supraorbital nerves at approximately 1.5 and 2.5 cm from the facial midline [9–11], before flap preparation at the supraorbital rim (Fig. 1)*.* Further fracture reduction and fixation or any other necessary procedures were performed by the primary author (P.P.) or by the same trainee who elevated the flap under close supervision of the first author. The scalp incision was closed in two layers using 2–0 sutures (Vicryl™, Ethicon, Johnson & Johnson Medical Devices, Norderstedt, Germany) for the galeal and subcutaneous tissues, and skin staples (Precise™ Vista Skin Stapler, 3 M Medica Abteilung Medical, Neuss, Germany). Before wound closure, two high-vacuum Redon drains were placed under both flap sides at the temporal/retroauricular region for 2–3 days. Skin staples were removed on 7 days postsurgery [8].

Although a study on 702 operations in 577 patients demonstrated that bandaging incisional scalp wounds after cranial neurosurgery did not add any benefit [12], we prefer using the head bandage to minimize the formation of large hematoma, and probably, subsequent infection, especially fractures in clean-contaminated areas, e.g., involvement of paranasal sinuses. An on-top soft dressing using an elastic bandage or kinesiologic taping was used to improve blood and lymph flow, remove congestion of lymphatic fluid or hemorrhage, and reduce postoperative pain and swelling (and trismus, if the masticatory organs are involved) [13, 14]. A Turban-style or Barton bandage was applied using gauzes, large laparotomy pads, and circumferential wrapping with a self-adhesive elastic bandage (Gazofix ™, BSN Medical GmBH, Hamburg, Germany). The bandage was removed with the Redon drains.

According to the current German Guideline [15], antibiotic prophylaxis with intravenous ampicillin/sulbactam (or clindamycin, if penicillin allergy) was given throughout hospital stay. Oral antibiotics were prescribed after hospital discharge on an individual basis. Postoperative pain management in accordance with the World Health Organization’s Pain Ladder included 1) around-the-clock intravenous paracetamol and/or metamizole, 2) as-needed subcutaneous piritramide and/or oral oxycondone, and 3) patient-controlled opioids or pain specialist consultation [16]. Pain management outcomes are beyond the scope of this study.

### Study Variables

The predictor variable was specialty background (OMFS/CFPS-Rs *vs.* TS-Rs). The main outcome variables were LHS and CFRCs until postoperative month six. The data were collected from surgical and/or intensive medical records in both electronic and handwriting forms. CFRCs were classified into seven parameters: (1) large incision or hypertrophic scar ≥ five mm width in dorsoventral direction, (2) hair loss, (3) sensory deficit, (4) facial motor deficit, (5) temporal fossa depression, (6) hematoma or prolonged bleeding, and (7) infections requiring prolonged antibiotics > one weeks. All of these seven parameters were recorded in binary (yes/no). The necessity or patient’s demand for plate and screw removal after midfacial and/or mandibular fracture repair, which were concomitantly performed with upper facial fractures, were not regarded as a complication, if this material removal was performed after six months since the fracture repair.

Other study variables were grouped into three groups: (1) demographic (age and gender of patients and trainees, clinical experience of trainees defined as years in practice), (2) anatomic (fracture type: upper facial fractures only *vs.* combined upper and midfacial fractures *vs.* panfacial fractures including a mandibular fracture), (3) therapeutic (operative time [OT; from the incision started to complete exposure of the fracture before bony reduction and fixation began], readmission rates [RR], and emergency room visits after the discharge [ERV] related to CFRCs or any other medically unwanted events).

### Data Management and Statistical Analysis

Data were collected and entered into a Microsoft Excel 2007 worksheet (Microsoft Inc., WA, USA), and statistical software MedCalc® (MedCalc Software Ltd., Ostend, Belgium) for analysis. Descriptive and binary statistics were compiled to provide an overview of the sample and identify significant outcome differences between both trainee groups (OMFS/CFPS-Rs *vs.* TS-Rs). Parameters associated with the outcomes of interest in bivariate analysis (*P* < 0.15) were included in a multiple logistic regression model. For all analyses, a *P* ≤ 0.05 was considered statistically significant.

To identify the benefit-risk profile of the surgery by OMFS/CFPS-Rs over the surgery by TS-Rs, we computed the number needed to treat (NNT, i.e., LHS < nine days [median]), to harm (NNH, i.e., overall CFRCs), and likelihood to be helped or harmed (LHH) as appropriate.

## Results

The study cohort comprised 5 trainees in each group (20% females). The trainee’s average age was 33.1 ± 3.2 years (range, 29–38), and the average years of practice since medical graduation were 4.7 ± 1.4 years (range, 3–8). During the study period, 71 of 97 patients undergoing the coronal flap for CMF trauma (19.7% women; average age, 40.2 ± 15.2 years; 46.5% treated by TS-Rs; 38% had combined upper and midfacial fractures) met the inclusion criteria. The excluded patients were operated by the primary author (P.P.), or by a trainee with enormous help from the first author. Patient’s demographic and anatomic, and trainee’s demographic parameters, as well as OT did not differ significantly between the two groups (*P* > 0.05) (Tables 1 and 2).

**Table 1:** Demographic and anatomical characteristics grouped by education background presenting descriptive and bivariate statistics

Characteristics	Overall	OMFS/CFPS-Rs	TS-Rs	*P* value (adjusted OR^§^ or RR^*^; 95% CI)
*Demographic*				
Patient’s sample size (%)	71 (100)	38 (53.5)	33 (46.5)	N/A
Patient’s age (years; mean ± SD [range])	40.2 ± 15.2 (17–79)	41 ± 15.4 (17–69)	39.3 ± 15.4 (18–79)	.65 (N/A; -5.62 to 9.01)
Median Patient’s age (years)	39	39.5	38	.53 (N/A; 457.08 to .025)
Female patient (%)	14 (19.7)	8 (21.1)	6 (18.2)	1.0 (1.2; .37 to 3.9)
Trainee’s sample size (%)	10 (100)	5 (50)	5 (50)	N/A
Trainee’s age (years; mean ± SD [range])	33.1 ± 3.2 (28–38)	34.8 ± 1.8 (33–37)	31.4 ± 4 (28–38)	.12 (N/A; -1.1 to 7.9)
Female trainee (%)	2 (20)	1 (120)	1 (20)	1.0 (1.0; .05 to 22.18)
Trainee’s years in practice (mean ± SD [range])	4.7 ± 1.4 (3–8)	4.2 ± 0.8 (3–5)	5.2 ± 1.9 (3–8)	.32 (N/A; -3.16 to 1.16)
*Anatomical*				
Upper facial fractures only (%)	20 (28.2)	11 (28.9)	9 (27.3)	1.0 (1.09; .38 to 3.07)
Upper and middle facial fractures (%)	27 (38.0)	17 (44.7)	10 (30.3)	.23 (1.86; .7 to 4.96)

*OMFS/CFPS-Rs* oral-maxillofacial or craniofacial plastic surgery residents; *TS-Rs* trauma surgery residents; *OR* odds ratio; *RR* relative risk; *N/A* not applicable

^§^adjusted to be binary before entering analyses

**Table 2:** Study outcomes grouped by education background presenting descriptive and bivariate statistics and benefit-risk appraisal

Characteristics	Overall	OMFS/CFPS-Rs	TS-Rs	*P* value (adjusted OR^§^ or RR^*^; 95% CI)	Absolute risk in percentage	NNT or NNH(95% CI)
*Therapeutic*						
OT (min. mean ± SD [range])	20.7 ± 5.3 (12–35)	19.8 ± 3.8 (14–30)	21.8 ± 6.6 (12–35)	.12 (N/A; -4.49 to .55)	N/A	N/A
LHS (days; mean ± SD [range])	9.2 ± 2.2 (6–15)	8.9 ± 2.2 (6–13)	9.5 ± 2.2 (7–15)	.24 (N/A; -1.67 to .42)	N/A	N/A
Median LHS (days)	9	9	9			
LHs ≥ 9 days^§^ (%)	40 (56.3)	21 (55.3)	19 (57.6)	.84 (.96^*^; .64 to 1.45)	( +)2.31 (− 20.8 to 25.4)	44 (3.9 to 4.8)
Patient with readmission (%)	2 (2.8)	1 (2.6)	1 (3.0)	1.0 (.86; .05 to 14.39)	N/A	N/A
Patient with emergency room visits (%)	3 (4.2)	2 (5.3)	1 (3.0)	1.0 (1.78; .15 to 20.54)	N/A	N/A
*Coronal flap-related complications*						
Visible/unfavorable or hypertrophic scar^†^ (%)	12 (16.9)	7 (18.4)	5 (15.2)	.76 (1.26; .36 to 4.44)	N/A	N/A
Hair loss^†^ (%)	10 (14.1)	6 (15.8)	4 (12.1)	.74 (1.36; .35 to 5.3)	N/A	N/A
Sensory deficit (%)	3 (4.2)	2 (5.3)	1 (3.0)	1.0 (1.78; .15 to 20.54)	N/A	N/A
Frontal nerve motor deficit (%)	0	0	0	1.0 (N/A)	N/A	N/A
Temporal fossa depression (%)	2 (2.8)	1 (2.6)	1 (3.0)	1.0 (.86; .05 to 14.39)	N/A	N/A
Hematoma^†^ (%)	3 (4.2)	2 (5.3)	1 (3.0)	1.0 (1.78; .15 to 20.54)	N/A	N/A
Infection^†^ (%)	2 (2.8)	1 (2.6)	1 (3.0)	1.0 (.86; .05 to 14.39)	N/A	N/A
Overall CFRCs^∆^ (%)	20 (28.2)	12 (31.6)	8 (24.2)	.5 (1.3^*^; .61 to 2.8)	( +)7.34 (− 13.45 to 28.13)	14 (3.6 to 7.4)

*OT* operative time; *LHS* length of hospital stay; *OMFS/CFPS-Rs* oral-maxillofacial or craniofacial plastic surgery residents; *TS-Rs* trauma surgery residents; *OR* odds ratio; RR – relative risk; *N/A* not applicable; *NNT* number needed to treat; *NNH* number needed to harm

^§^ adjusted to be binary before entering analyses. ^†^ All postoperative infections occurred after huge hematoma formation, and all hair losses resulted from scars (i.e., for calculation of overall complications, we therefore counted hematoma with infection as 1 case, and scar with hair loss as 1 case). ^∆^ Overall CFRCs were counted by subjects with at least one of the seven complications

Patients undergoing flap raising by TS-Rs experienced neither longer LHS (*P* = 0.24; 95% CI, − 1.67 to 0.42) nor more RR (*P* = 1.0; adjusted odds ratio [OR_adj_], 0.86; 95% CI, 0.05 to 14.39) nor more ERV (*P* = 1.0; OR_adj_, 1.78; 95% CI, 0.15 to 20.54) nor higher complications (*P* = 0.6; OR_adj_, 1.44; 95% CI, 0.5. to 4.12) than those operated by OMFS/CFPS-Rs. 60% of the CFRCs observed were visible/unfavorable or hypertrophic scar with/without hair loss (Table 2)*.*

Regarding the benefit-risk appraisal, approximately one in every 44 patients will benefit from the surgery by OMFS/CFPS-Rs in terms of shorter LHS (< nine days; NNT = 44 [95% CI, 3.9 to 4.8]), and approximately one in every 14 patients will be harmed from CFRCs after surgery by OMFS/CFPS-Rs (NNH = 14 [95% CI, 3.6 to 7.4]), when compared to surgery by TS-Rs until postoperative month six. The resultant LHH is 0.32, discarding the necessity of coronal flap raising by OMFS/CFPS-Rs. In other words, TS-Rs, when assisted in ≥ two surgeries, can operate the coronal flap as well as OMFS/CFPS-Rs did (Table 2).

## Discussion

Similar to most European countries, CMF surgery in Germany is the core specialty responsible for CMF injuries. Its unique characteristics comprise undergraduate dual-degree (MD *plus* DDS/DMD) requirements, five-year training standard, and full scopes of head and neck surgical practice [17, 18]. However, only 158 of 1914 (or 8.3%) German hospitals can offer CMF patient care [3, 4]. There has, therefore, been an upward trend in hiring one or two, either full- or part-time, CMF surgeons in the trauma unit of hospitals without a standalone CMF section. This circumstance has compelled many changes and challenges, i.e., CMF education is provided for other specialty trainees, while it is unapproved and unaccredited by the German Medical Council in all federal states. Rotational trainee sharing, hence, becomes essential in this organization model, and requires investigations concerning the benefit-risk appraisal including patient safety.

The key aim of this study was to determine whether trainees’ background influenced patient outcomes with regard to LHS and CFRCs. We hypothesized no outcome differences between the trainee groups. The results of this study accepted the null hypothesis, i.e., the coronal flap could be performed by TS-Rs as the primary surgeon with favorable clinical outcomes, after they had assisted in ≥ two coronal flaps performed by the skilled CMF surgeon. This finding suggests a steep (easy) learning curve of the procedure, i.e., an uncomplicated, non-technically demanding procedure. Experienced trainees in other surgical specialties can begin CMF trauma surgery in some circumstances (for example, when the CMF consultant surgeon is busy with other patient care) and may need the consultant only for skeletal reduction and fixation, and dental occlusion control, if they are properly trained. Our findings appear comparable to those of other works reported by staff surgeons in diverse specialties (Table 3), suggesting that well-trained trainees could operate this flap so well as staff surgeons do.

**Table 3:** Outcome comparison with previously relevant publications regarding coronal approach to craniofacial fractures.

Authors (publication year, country of origin)	Operator’s factors, i.e., level of career, and specialty	Sample size (sample size per year)	OT (min.)	*P* value (95% CI)	LHS (days)	*P* value (95% CI)	CFRCs (%)	*P* value (adjusted OR; 95% CI)
Pitak-Arnnop et al*.* (the present study, Germany)	Residents, [dual-degree] OMFS, or CFPS	38 (19)	19.8 ± 3.8 ^a^ (14–30)	.12 (− 4.5 to .6) ^a *vs.* b^	8.9 ± 2.2 ^d^ (6–13)	.24 (− 1.7 to .4) ^d *vs* e^	12 (31.6) ^g^	.49 (.7; .2 to 2) ^g *vs.*h^
	Residents/fellows. trauma surgery	33 (16.5)	21.8 ± 6.6 ^b^ (12–35)		9.5 ± 2.2 ^e^ (7–15)		8 (24.2) ^h^	
	Pooled: both trainee groups	71 (35.5)	20.7 ± 5.3 ^c^ (12–35)		9.2 ± 2.2 ^f^ (6–15)		20 (28.2) ^i^	
Sikkerimath et al*.* [19] (2021, India)	Staffs, [single-degree/BDS] OMFS	40 (8)	?	N/A	?	N/A	15 (37.5)	.58 (.8; .3 to 2) ^*vs.* g^ .23 (.5; .19 to 1.5) ^*vs.*h^ .3 (.7; .3 to 1.5) ^*vs.* i^
Kumar et al*.* [20] (2016, India)	Staffs, [single-degree/BDS] OMFS	10 (?)	34 ± 7.7 (25–45)	** < .0001 (-17.6 to **− **10.8) **^***vs.* a**^** < .0001 (-17.2 to -7.2) **^***vs.* b**^** < .0001 (**− **17.1 to -9.) **^***vs.* c**^	3.5 ± .5 (3–4)	** < .0001 (4 to 6.8) **^***vs.* d**^ ** < .0001 (4.6 to 7.4) **^***vs.* e**^ ** < .0001 (4.3 to 7.1) **^***vs.* f**^	1 (10)	.2 (4.2; .5 to 36.6) ^*vs.* g^ .35 (2.8; .3 to 26.4) ^*vs.*h^ .25 (3.5; .4 to 29.7) ^*vs.* i^
Rajmohan et al*.* [1] (2015, India)	Staffs, [single-degree/BDS] OMFS	6 (?)	12 ± 1 (10–13)	N/A	?	N/A	1 (16.7)	.47 (2.3; .3 to 2) ^*vs.* g^ .7 (1.6; .16 to 15.8) ^*vs.*h^ .55 (2; .2 to 17.8) ^*vs.* i^
Gabrielli et al*.* [21] (2012, Brazil)	Staffs, [single-degree/DDS] OMFS	132 (6.9)	?	N/A	?	N/A	63 (47.7)	.08 (.5; .24 to 1.1) ^*vs.* g^ **.02 (.35; .15 to .83) **^***vs.*h**^ **.008 (.53; .23 to .8) **^***vs.* i**^
Kim et al*.* [22] (2012; South Korea)	Staffs, plastic surgery	8 (2.7)	?	N/A	?	N/A	5 (62.5)	.1 (.3; .06 to .14) ^*vs.* g^** .048 (.2; .04 to .99) **^***vs.* h**^ .06 (.24; .05 to 1.1) ^*vs.* i^
Shetty et al*.* [23] (2009, India)	Staffs, [single-degree/BDS] OMFS	12 (3)	28.7 ± ? (25–40)	N/A	?	N/A	2 (16.7)	.3 (2.3; .4 to 12.2) ^*vs.* g^ .6 (1.6; .3 to 8.9) ^*vs.* h^ .4 (2; .4 to 9.7) ^*vs.* i^
Kerawala et al*.* [24] (2000, UK)	Staffs, [dual-degree] OMFS	68 (11.3)	?	N/A	?	N/A	12 (17.6)	.1 (2.2; .9 to 5.4) ^*vs.* g^ .4 (1.5; .5 to 4.1) ^*vs.*h^ .14 (1.8; .8 to 4.1) ^*vs.* i^
Kiyokawa et al*.* [25] (1999; Japan)	Staffs, plastic and neurosurgery	18 (?)	?	N/A	?	N/A	1 (5.6)	.06 (7.8; .9 to 66) ^*vs.* g^ .13 (5.4; .6 to 47.6) ^*vs.* h^ .07 (6.7; .8 to 53.5) ^*vs.* i^
Nestle et al*.* [26] (1998; Germany)	Staffs, [dual-degree] OMFS	59 (8.4)	?	N/A	?	N/A	20 (33.9)	.8 (.9; .4 to 2) ^*vs.* g^ .3 (.6; .24 to 1.6) ^*vs.*h^ .48 (.76; .36 to 1.6) ^*vs.* i^
Cheney et al*.* [27] (1995; USA)	Staffs, otolaryngology	14 (4.6)	?	N/A	6.7 ± 4 (?)	**.006 (.8 to 4.2) **^***vs.* d**^ **.003 (1 to 4.6) **^***vs.* e**^ **.001 (1 to 4) **^***vs.* f**^	2 (14.3)	.2 (2.8; .5 to 14.4) ^*vs.* g^ .45 (1.9; .35 to 10.5) ^*vs.*h^ .3 (2.4; .48 to 11.5) ^*vs.* i^
Frodel & Marentette [6] (1993; USA)	Staffs, otolaryngology	101 (?)	?	N/A	?	N/A	42 (41.6)	.28 (.65; .3 to 1.4) ^*vs.* g^ .08 (.45; .2 to 1.1) ^*vs.*h^ .07 (.55; .3 to 1.1) ^*vs.* i^
Mitchell et al*.* [28] (1993, UK)	Staffs, [dual-degree] OMFS	50 (10)	?	N/A	?	N/A	19 (38)	.5 (.75; .3 to 1.8) ^*vs.* g^ .2 (.5; .2 to 1.4) ^*vs.*h^ .26 (.64; .3 to 1.4) ^*vs.* i^
Abubaker et al*.* [29] (1990, USA)	Staffs, [single-degree/DMD] OMFS	27 (9)	?	N/A	?	N/A	3 (11.1)	.06 (3.7; .9 to 14.7) ^*vs.* g^ .2 (2.6; .6 to 10.8) ^*vs.*h^ .09 (3.1; .85 to 11.6) ^*vs.* i^
Shepherd et al*.* [30] (1985, UK)	Staffs, [dual-degree] OMFS	24 (?)	?	N/A	?	N/A	18 (75)	**.001 (.15; .05 to .5) **^***vs.* g**^ **.0003 (.1; .03 to .4) **^***vs.*h**^ **.002 (.13; .05 to .4) **^***vs.* i**^

Studies without adequate information on operative time or length of hospital stay or CFCR and those presented as a small case series (*n* < 5) were excluded. Continuous data are listed as mean ± SD (range). Categorical data are presented as number (percentage)

*OT* operative time; *LHS* length of hospital stay; *OMFS* oral-maxillofacial surgery; *CFPS* craniofacial plastic surgery; *BDS or DDS or DMD* a dental degree; *N/A* not applicable or not computable;–data not shown

Statistically significant *P*-values are indicated in **bold** typeface

German medical education, especially residency training, remains highly variable. Previously, residents in this country were autodidactic, i.e., they could independently work from their mentors, i.e., staffs (“Facharzt/-ärztin”) and consultants (“Oberarzt/-ärztin”), and the departmental head. Nowadays, it is mandatory that at least one staff or consultant surgeon is present during major surgeries. Certain patient care such as minor surgeries and patient follow-ups may not require continuous guidance or supervision, if respective/related competencies have been achieved and reassured [31, 32]. The self-standing practice simply facilitates case-based learning, and in surgery, promotes operative skills, and encourages trainees to participate in a discussion, where relevant, with their mentors. This “learning by mentorship” format not only assures learner’s engagement, but adds deliberative tools, such as critical analysis, appraisal and problem solving to an individual patient. Moreover, it provides a practical application of theoretical knowledge passively gained from earlier lectures and seminars, and the cultivation of professionalism, i.e., confidentiality, competency and responsibility [33]. However, close mentorship was found to be linked to better resident’s satisfaction [32, 33]. A recent German survey showed very low satisfaction among otolaryngologic residents due to training quality, i.e., < 30% of residents were satisfied or very satisfied. The dissatisfaction mostly arose from limited supervision and low numbers of surgery cases [32]. It can therefore be implied that surgical educators must find the “middle path” to ameliorate the training quality.

In the real world, teaching surgeons are often pressured toward operating more and teaching less. Possible reasons are diminishing reimbursements, demanding high-quality outcomes including low complication rates and short LHS, and inefficiency of regulation and defensive practice. All pressures trample over surgical education and learner’s satisfaction [34]. The link between intraoperative independence (self-standing performance) and trainee’s satisfaction is beyond this study’s scope and requires further investigations.

One provoking factor is that too many residents, despite reduced workloads of the faculty surgeons, concurrently reduce operative volume for trainees. Apart from preference (e.g., toward facial aesthetic surgery for further career in private practice [35]), the perception of cases being “stolen” by one another may develop and subsequently curtails satisfaction of residents in training.

At the training end, residents and fellows are supposed to enter the exit examination. Surgical board certification examinations in Germany are unlikely to be based on practical competencies, but depend mainly on the examiners (i.e., neither rigid standard nor writing examination nor case presentation), and focus on the minimal number (“not” the quality) of performed procedures. It was the first author (P.P.)’s subjective experiences that some CMF surgeons were be able to pass the German board examination without an essential ability to perform basic procedures (i.e., without the need for help), for example, tracheostomy, orbital floor fracture repair, neck dissection, or extraoral draining of deep space infections of the neck. A possible explanation is that some CMF departments arrange very long ward works, for example, one CMF center in Saxony arranged a six-month ward rotation (with no involvement of surgery at all) for its trainees, which reduces the trainee’s operative skill and confidence during this long period of ward rotation. Some centers separated their trainees into subgroups, e.g., trainees in one CMF clinic in Hessen are divided into cleft-craniofacial and orthognathic, or head and neck tumor and reconstructive, or CMF trauma subgroups, according to the trainee’s interest and/or department’s organization. No or at least no wider competency across subgroups is gained and maintained and appears problematic. Trainees in the cleft-craniofacial and orthognathic subgroup often face difficulty in post-microsurgical monitoring in oncologic reconstructive patients, when the trainees work during the night shift, and trainees in the CMF trauma subgroup almost never perform a neck dissection by themselves (unpublished data).

During the 14th International Congress of the Turkish Association of OMF/CMF surgeons in 2007, Daniel M. Laskin [36], a former professor of Virginia Commonwealth University, VA, USA *(deceased)*, presented the CMF scopes of practice in three levels: (1) areas of expertise, which are oral pathology/medicine (including basic knowledge in oral mucosal diseases, temporomandibular joint [TMJ] disorders and orofacial pain/headache, and CMF radiology), dentoalveolar and preprosthetic surgery (including dental implantology), and CMF traumatology, (2) areas of competence involving orthognathic surgery, TMJ surgery, and local reconstructive surgery, and (3) area of familiarity consisting of cleft-craniofacial surgery, regional reconstructive surgery, oncologic surgery, and esthetic surgery [36]. Because OMF/CMF surgery outside Europe is dentally based and often located in dental schools, it is unknown whether the Laskin’s scope classification is applicable to medically based CMF surgery in Europe. We refer interested readers to the complete lists of German CMF surgeons’ tasks detailed by Pitak-Arnnop [4, 18] and Pitak-Arnnop et al*.*[17]

To promote the “drilling warriors for future battlefields” in German surgical training, the “surgical sub-step” concept has been developed. This concept acquires surgical skills as a part of more complex operations that might be too difficult for trainees at the time, e.g., tracheostomy by a second-or-third-year resident, and vascular anastomosis by fourth-or-fifth-year trainees during a complex head and neck reconstruction after extensive tumor resection. The concept can be easily implemented in a surgical department based on an individual basis and increases resident’s satisfaction [37].

DaRosa et al*.* [38] described that the levels of teaching practical surgical skills should advance from 1) “assisting” (show and tell stage) to (2) “smart help,” to (3)“dumb help” (i.e., I can do *all parts* of the surgery, albeit with some hesitation or even floundering, but requiring only “passive” assistance from the consultant; consistent with the Peak of “Mount Stupid” in the Dunning-Kruger effect curve [the first author, P.P.,’s interpretation]), and (4) finally to “performing alone”. Surgical sub-steps (apart from wound closure and bandaging, which are common sub-steps for trainees and medical students) can be adapted to all of these levels and open possibility to gain competency before advancing to self-performing the whole surgery. The German Young Surgeons Working Group („Chirurgische Arbeitsgemeinschaft „Junge Chirurgen “, CAJC) currently plans the continuous sub-step registry in order to improve training quality and measure effects of the awareness campaigns. With this continuous registry, the proposed sub-step concept will hopefully be applied more often in the operating theater in this country [37].

Last but not least, the high percentage of males over females (M:F) in this study can result from the selection bias, or the real-world ratio of M:F in surgery, or both. Statistically, the ratios of M:F consultant surgeons in the UK and overall surgeons in Germany were found to be only ~ 8:1 and ~ 3.5:1, respectively (available from https://www.rcseng.ac.uk/careers-in-surgery/women-in-surgery/; https://www.derstandard.de/story/2000134267964/studie-zu-gender-effekt-maenner-chirurgie-fuer-patientinnen-gefaehrlich; accessed on November 29, 2022). Some of us (A.N., K.S., N.S., P.P.) found the meaningful associations between female CMF surgeons and low scientific productivity and limited progress in academic surgical career [39]. Women in CMF surgery is therefore an important issue for future research.

Prior studies often compared outcomes between surgeries performed by skilled staff surgeons *vs*. those by trainees [5, 34]. The strength of our study is, thereby, the use of objective measures to evaluate outcomes using the coronal flap as a part of CMF injury patient care to argue for or against the “surgical sub-unit” learning concept. This study has, however, limitations which merit discussion. First, its retrospective design hampers identification of clinical-/operator-related factors that could affect the treatment outcome. Second, possible selection bias may exist, i.e., relatively easy cases were assigned to trauma trainees, leading to favorable outcomes. Third, high volume of CMF injuries could increase trainees’ skills and success rate of the surgery, albeit performed by novice trainees. Accumulation of clinical experience at hospitals with high patient volume, taught by a skilled, qualified surgeon, may reduce the learning curve in surgery, and probably, interfere the study’s generalization or external validity especially in a small trainee cohort (n = 5 in each study arm) [5]. Fourth, the post hoc power based on CFRCs was < 50%, suggesting the high possibility of the beta error. However, to reach the post hoc power of 100, we would have needed the 11-fold increased sample size, i.e., in a 22-year interval of data collection. Multicentric studies could exponentially increase the sample size, but inter-operator variables such as surgeon’s skill and experiences could be problematic, and the study would have suffered from poorer internal validity instead. Compared to previous studies in the literature, 97 coronal flaps for CMF trauma in two years, indeed, indicate the very high hospital volume. Moreover, statistical significance by intentional adjustment of the sample size is considered as scientific misconduct [40].

## Conclusions

Surgical outcomes regarding coronal flap raising were similar between the OMFS/CFPS-Rs and TS-Rs groups. Hence, participation of TS-Rs in CMF trauma surgery appears possible and suitable without compromising patient safety. Albeit not statistically significant, the overall CFRC rates after surgery by OMFS/CFPS-Rs preceded those by TS-Rs. Our results encourage the striking trend of including a CMF surgeon in the trauma department of general hospitals (where there is no OMF department). In other words, these findings can be a double-edged sword. As the core specialty responsible for CMF trauma patient care, the CMF training system needs to improve multiple aspects for its OMFS/CFPS-Rs, e.g., more opportunity of operative autonomy, and experience. CMF trainees are expected to do better coronal flap surgery than TS-Rs.

## Data Availability

Deidentified individual participant data are not available. Based on the current patient data protection law in Germany, open access to the raw data is not allowed. The datasets generated and analyzed during this study are available from the corresponding author upon reasonable request.

## Abbreviations

ASA: The American Society of Anesthesiologists
CFRC: Coronal flap-related complications
CMF: Craniomaxillofacial (injury or surgery discipline)
ERV: Emergency room visits after discharge
ICMJE: International Committee of Medical Journal Editors
LHH: Likelihood to be helped or harmed
LHS: Length of hospital stay
MH: Main hospital
MF: (The ratio of) males to females
NNH: Number needed to harm
NNT: Number needed to treat
OCEBM: The Oxford Centre for Evidence-Based Medicine
OMF: Oral-maxillofacial (surgery discipline)
OMFS/CFPS-Rs: Oral-maxillofacial or craniofacial plastic surgery residents
OR_adj_: Adjusted odds ratio
OT: Operative time (i.e., incision start to complete exposure of the fracture)
PGY: Postgraduate year
RR: Readmission rates; relative risk
STROBE: The STrengthening the Reporting of OBservational studies in Epidemiology
TMJ: Temporomandibular joint
TS-Rs: Trauma surgery residents
95% CI: 95% Confidence interval
